# The Old Mill

**Published:** 1912-11-02

**Authors:** 


### HOSPITAL WEAKNESS AND HOSPITAL STRENGTH.

### I.—The Old Mill.

"Every man to his last" is a good maxim for workers who mean business. But what is a man's last? Take, for instance, the position of the superintendent or secretary or the chief officials, medical and lay, who devote their best energies to the work of a particular hospital. No doubt it is the privilege of every such worker to make his first interest and his first work the accomplishment of everything within his means which is best calculated to bring success to the institution with which he is associated. This principle is sound and good, but it is too limited if the workers in question are individually and collectively to attain to the knowledge, proficiency, and success which lie open to every one of them. Every hospital, and especially every voluntary hospital, is but a unit in a great system of medical relief and hospital work. The heart and motive-power of such a system must be efficiency. Efficiency cannot be obtained without knowledge, and knowledge, in a hospital dependent upon voluntary contributions, is the magnet which most certainly attracts and secures the money necessary to make an individual hospital of this type successful, by keeping it up to date and so winning new supporters every day.

### Slowness to Take New Ideas.

Business men in the United States often laugh, and with reason, at the slowness to assimilate new ideas which too often pervades the conduct of great businesses in Great Britain. This danger to the whole commonwealth in this country is fortunately being lessened every day by the necessity which compels the more alert of our business-men in England to realise that, if they are to earn dividends they must spend money, and spend it prodigally too, in the present day. Apply the same principle to the responsible heads of all departments of a voluntary hospital, where the raising of the necessary funds—that is, the payment of dividends by developing the hospital and maintaining it at the highest state of efficiency—means the possession and wise application of up-to-date knowledge every day. The active working of the British Hospitals Association and of the Association of Hospital Officers is a good sign, for each of these bodies to succeed must be so organised as to disseminate knowledge and the enforcement of the highest conduct in institutional affairs. They have enlisted an increasing number of members to join their ranks during the last few years, and there are signs that some of the officers referred to are beginning to realise that to be efficient, they must keep their eye upon the trend of events and the changes which are impending, for both must affect their work for hospitals in many ways. How far these facts may result in good or evil to a particular hospital, or to the whole voluntary system of hospitals, must depend largely upon the awakening of the responsible administrators of these great institutions to the necessity of giving time and thought to the acquirement of knowledge bearing upon their work, which can alone be obtained by the study of what is passing outside their immediate field of labour.

### Knowledge and its Refreshment.

We are led to these reflections and the enforcement of these principles by the knowledge that the very basis on which the Government may and probably will act in providing hospital treatment for the 15,000,000 insured persons, for whom they must provide it on and after January 15, 1913, has already been promulgated and is available for the study and information of all who have eyes to see and minds to study, and understand these important questions. The Hospital has dealt with hospital questions week by week for upwards of twenty-six years. The bound copies of The Hospital constitute a library of information and contain all the material facts and documents connected with the development, expansion, and past and present position of the voluntary hospitals in this country. The most active minds in the hospital world have knowledge of the fact and keep The Hospital filed in their offices for constant reference, many of them have assured us with gratitude from time to time. In Germany for eight years past every week there has been published the *Zeitschrift für Krankenanstalten*, which fulfils the same purpose in Germany that The Hospital fulfils for the British Empire. In Germany every responsible hospital manager files our German contemporary, and once a year is published a diary which contains an accurate summary of the material facts and information contained in the *Zeitschrift für Krankenanstalten*, which every governor of every hospital in Germany keeps on his desk. We found that the attitude of the great body of hospital managers in each country towards these two technical journals illustrates two entirely different states of mind. The German hospital officers say they must have information; they must know what is going on; they must learn how to meet the financial and other difficulties which they have continually to face; and so they not only take but are diligent students of the *Zeitschrift für Krankenanstalten*. In Great Britain, as we have said, not all but only the more active and responsible of the chief officials of hospitals, medical and lay, take and study The Hospital because they recognise, as our contemporaries recognise, that whereas we seldom touch treatment, we undoubtedly do supply the first information and the soundest knowledge concerning questions of administrative medicine and administration generally.

### A Wider Outlook Essential.

No doubt a hospital superintendent or secretary and each of the other heads of departments of a great voluntary hospital have much to occupy their time and thought, but to be efficient they should take a leaf out of the German's book and the American's book, and be careful to devote a little time each week to the acquirement of a knowledge of what is going on outside their own walls in the hospital world. All hospital managers are naturally disturbed by the effects which may follow the passing of the National Insurance Act. THE HOSPITAL during the last two years has dealt with these questions in a statesmanlike way, and has republished a series of articles which contain the great principles and a sound plan for the solution of the problem upon a basis which will maintain the voluntary system and place it upon a sounder foundation than it has ever yet secured. Yet we find as an outcome of the publication of a series of articles on the hospital system of Germany, now running week by week in THE HOSPITAL, that the large majority of the responsible managers and chief officials of the hospitals in Great Britain have not a knowledge of these sources of information or the contents of the pamphlets referred to. Nevertheless, it is literally the fact that those pamphlets, as the immediate future is likely to prove, contain the basis upon which the hospital system of this country will be reorganised and made stronger than ever before in its history.

WAKE UP, COMMITTEEMEN AND OFFICIALS!

Wake up, therefore, hospital officials and hospital managers; learn while there is time what are the lines of least resistance and how the voluntary hospitals can be made secure; learn, too, that by co-operating with the State upon strong lines which maintain the voluntary principle intact, you can remove from the voluntary hospital all existing abuses, and provide it with all the money required to make it absolutely efficient in every department, as explained in the columns of THE HOSPITAL, and will yet further be explained in the course of the next few weeks in the series of reports referred to. Thus you can be ready to bring knowledge to bear upon every emergency which arises and be prepared to solve it upon a basis which must prove the consolidation of the voluntary system. The old mill was good enough to grind all the corn which under former conditions it was erected to crush. To-day we have entered upon a wider and vaster field of labour and responsibililty, and the responsible workers in every hospital who are worth their salt will bestir themselves to win for their institution the strength which a little industry on their part in the acquirement of knowledge must provide. Those voluntary hospitals which possess the most active-minded members of committees, by following the German example, can soon equip themselves with the necessary knowledge to test to what extent their staff are awake and efficient, and can so learn the standard of knowledge of modern hospital requirements which their chief officials possess.

